# A landscape analysis of leadership training in postgraduate medical education training programs at the University of Ottawa

**Published:** 2016-10-18

**Authors:** Marlon Danilewitz, Laurie McLean

**Affiliations:** 1 Faculty of Psychiatry, University of British Columbia; 2 Department of Otolaryngology, University of Ottawa

## Abstract

**Background:**

There is growing recognition of the importance of physician leadership in healthcare. At the same time, becoming an effective leader requires significant training. While educational opportunities for practicing physicians exist to develop their leadership skills, there is a paucity of leadership opportunities for post graduate trainees. In response to this gap, both the Royal College of Physicians and Surgeons of Canada and the Association of Faculties of Medicine of Canada have recommended that leadership training be considered a focus in Post Graduate Medical Education (PGME). However, post-graduate leadership curricula and opportunities in PGME training programs in Canada are not well described. The goal of this study was to determine the motivation for PGME leadership training, the opportunities available, and educational barriers experienced by PGME programs at the University of Ottawa.

**Methods:**

An electronic survey was distributed to all 70 PGME Program Directors (PDs) at the University of Ottawa. Two PDs were selected, based on strong leadership programs, for individual interviews.

**Results:**

The survey response rate was 55.7%. Seventy-seven percent of responding PDs reported resident participation in leadership training as being “important,” while only 37.8% of programs incorporated assessment of resident leadership knowledge and/or skills into their PGME program. Similarly, only 29.7% of responding residency programs offered chief resident leadership training.

**Conclusions:**

While there is strong recognition of the importance of training future physician leaders, the nature and design of PGME leadership training is highly variable. These data can be used to potentially inform future PGME leadership training curricula.

## Introduction

The current practice of medicine necessitates a diversity of leadership skills in order to comprehensively and efficiently respond to the complex needs and demands of the current and ever-evolving healthcare system. Although there are growing opportunities for leadership development for practicing physicians, many believe that leadership training should begin long before licensure occurs. In fact, the Association of Faculties of Medicine of Canada (AFMC), in their documents titled “The Future of Medical Education in Canada (Undergraduate Medical Education and Postgraduate Medical Education)” recommended that leadership development be included in the curricula at both the Postgraduate Medical Education (PGME) and Undergraduate Medical Education (UME) levels.^1^,^2^ In particular, the development of leadership at the PGME level is included in the AFMC’s collective vision for graduate medical education as one of its 10 core recommendations, highlighting the need to develop a national core leadership curriculum to focus on “professional responsibilities, self-awareness, providing and receiving feedback, conflict resolution, change management, and working as part of a team as a leader, facilitator, or team member.”^3^

Similarly, the Royal College of Physicians and Surgeons of Canada (RCPSC) recently modified the former CanMEDS role of “manager” with the role of “leader”, highlighting the importance of strong leadership as a fundamental role in training future physicians. As part of the leadership role, the RCPSC describes four key competencies (Table 1).^4^

While the mandate to train leaders is clear, there has been little research to date exploring leadership training and/or curricula in PGME. Of the research studies to examine innovative leadership programs for postgraduate medical trainees, only a few have rigorously evaluated the interventions using quantitative measures.^5^

In addition to the paucity of research in the area of leadership interventions in PGME, little is known about the landscape of leadership opportunities and curricula at the individual program level. Interviewing program directors to assess current practices in leadership training would be the first critical step towards developing best practices in leadership curricula. The goal of this study was to determine the motivation for PGME leadership training, the opportunities available, and educational barriers experienced by PGME programs at the University of Ottawa. Perhaps by better understanding the current milieu of PGME leadership training at one institution, we can replicate this study at other sites and, in the future, develop better curricula.

## Methods

The study protocol was reviewed by the Ottawa Health Sciences Research Ethics Board and granted exemption.

### Quantitative

#### Generation of questionnaire items

Questionnaire items were generated through examination of the literature, discussions with experts in leadership development, four PGME Program Directors (PDs) with an interest in leadership development at the University of Ottawa, and the RCPSC CanMEDS Leadership role key competencies framework.^4^ The final survey was reached through consensus. The survey was reviewed by two faculty members, who are medical clinicians, and two medical students, all from the University of Ottawa. The final set of questions was obtained once consensus had been reached after nine iterations. The final piloted survey was designed with elements in keeping with Cochrane’s review on maximizing survey response rates.^6^ The survey was designed to address the following topics:

- Specialty program preparedness for CanMEDS 2015 leadership role key competencies
- Current leadership training/curricula opportunities
- Current challenges to implementing leadership training
- Potential strategies to improve leadership training
- Chief residency training opportunities
- Methods of evaluating leadership training
- The impact of leadership training in PGME

A Likert scale was used for quantitative questions. Free-text fields were used for open-ended questions to acquire qualitative data from PDs. An online version of the survey was developed using Fluid Survey™, a web-based software. A copy of the online survey can be found in Appendix 1.

#### Administration of the survey

All PDs (n=70) at the University of Ottawa were invited to participate.

The online Fluid Survey™ survey was sent via email to all 70 PDs, along with an email explaining the purpose and significance of the study. Respondents had the opportunity to reveal their identity or reply anonymously. To encourage completion of the survey, respondents were sent three email reminders at weekly intervals and were supplied with paper copies of the survey.

### Qualitative

#### Selective interviewing

PDs of PGME programs with strong models of integrating leadership training into resident education were invited to participate in a one-on-one interview to elicit further information about their program’s leadership training/curricula. Selection of strong models of leadership training was based on the number of hours dedicated to leadership training, leadership opportunities offered, and compliance with CanMEDS 2015 leadership role key competencies. Two PDs were interviewed. Semi structured interviews were carried out by the author (MD) which focused on obtaining a more comprehensive understanding of leadership training opportunities. Interviews averaged 30 minutes in duration. Field notes were taken. Questions addressed the following topics: leadership opportunities, leadership training for chief residents, assessment methods, process of leadership curriculum development, unsuccessful programs, and resident participation and feedback.

#### Analytic approaches

With respect to the online survey, quantitative data were analyzed for descriptive statistics using Fluid Surveys© and qualitative free-text responses were reviewed for themes. With respect to the semi-structured interviews, field notes were analyzed for themes and innovative ideas.

## Results

### Quantitative: online questionnaire analysis

**Figure 1:**
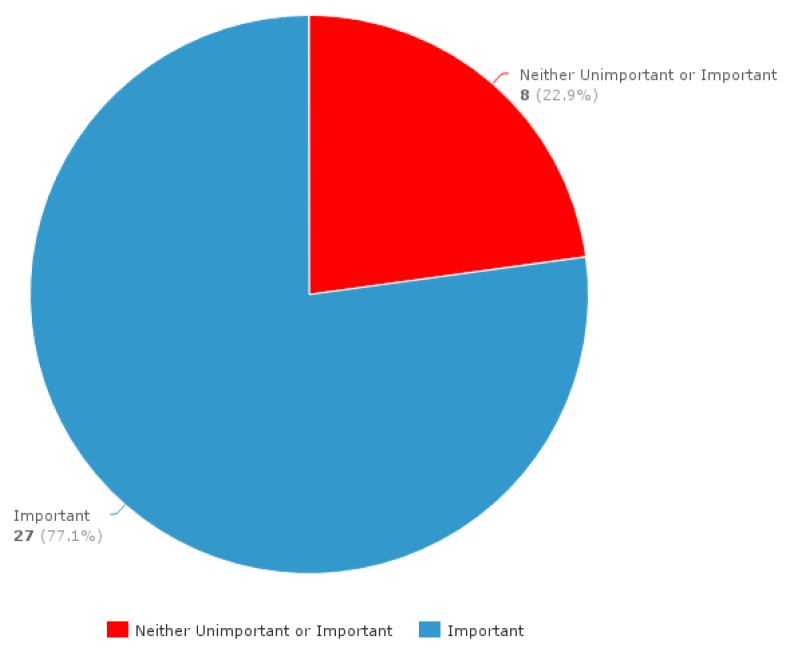
PD rating of level of importance of resident participation in leadership training (*n* = 35)

The survey response rate was 55.7% (n=39). Programs identified by the responding PDs, (n=32), are shown in Table 2 alongside the total number of trainees per program. The majority of PDs (77.1%, 27/35) reported resident participation in leadership training as being “important” on a three level Likert scale, ranging from “unimportant” to “important” (Figure 1).

Program preparedness for CanMEDS 2015 leadership role key competencies varied significantly between competencies. Competencies that PDs reported being “not prepared for” included: “Design and organize elements of healthcare delivery” (30.8%, 12/39), “Facilitate change in healthcare to enhance services and outcomes” (41.0%, 16/39), and “Manage health human resources in a practice” (30.8%, 12/39) (Table 2). Alternatively, the competency that PDs reported most consistently being “prepared” to address was, “Manage career planning” (61.5%, 24/) (Table 3).

The delivery of residency leadership programs varied greatly between programs. The majority of programs offered optional activities as part of their leadership program. The leadership activity that was most often made a mandatory component of leadership programs was participation in (a) multisource feedback (MSF) exercise(s) (mandatory in 86.8% of programs, 33/38, Table 4). MSF is considered part of leadership development as it attempts to address the self-awareness pillar of effective leadership.

Assessment of resident leadership was only undertaken by 37.8% (14/37) of responding programs. Of those programs that incorporated some mode of assessment, the most commonly used means of assessment was “direct observation”, the details of that encounter not further elucidated. With respect to leadership training, the majority of programs did not incorporate training that addressed self-reflection, self-management, or self-awareness themes (Table 5). Only 29.7% (n=11) of programs offered training for chief residents, while 54.1% (n=20) responded that they did not currently offer any training for chief residents, and 16.2% (n=6) of programs were uncertain as to whether any chief resident training was offered (Total response rate 37). Only 12 programs of 37 responding programs (32.4 %) reported conducting some form of formal assessment of chief residents activities, not necessarily specific to leadership qualities.

The most significant barriers to implementing leadership training, according to PDs, were scarcity of time (62.2%, 23/37), lack of knowledge of how to develop a leadership training curriculum (47.4%, n=18, Total Reponses = 38) and lack of or limited human resources (44.7%, 17/38) (Table 6). Alternatively, the following factors were considered to be “Not a Barrier” to implementing leadership training by a large percentage of PDs: lack of buy-in by residents (56.8%, 21/37), lack of evidence to support leadership training (57.1%, 20/35), and lack of buy-in by faculty (40.5%, 15/37) (Table 6).

PDs found the majority of listed supports to be “very helpful.” In particular, the specific supports of “Information about other implemented leadership training curricula” (70.3%, 26/37) and “Infrastructure to support leadership training” (74.3%, 26/35) were most consistently reported by PDs as being “very helpful.” Alternatively, further research was found to be the least helpful according to PDs; only 13.5% (5/37) of PDS reported it to be “very helpful,” 54.1% (20/37) reported it to be “somewhat helpful.” and 32.4% (12/37) reported it to be “not helpful” (Table 7).

### Qualitative

Several interesting ideas emerged from the semi-structured interviews with the two PDs and the comments obtained from free text answers of the survey questionnaire. Eight themes emerged (See Table 8 in Appendix 2):

1. Importance of leadership training
2. Need for support for leadership training
3. Need for access to leadership training opportunities at national/international level(s)
4. Need for in-situ PGME leadership opportunities at home institution
5. University stewardship for PGME leadership training
6. Redefine service to leadership
7. Information sharing
8. Recognition for PGME trainees who excel in leadership

## Discussion

The AFMC and the RCPSC have both stated that leadership training for the PGME trainee is important. This message appears to have been heard with 77.1% (n=27, Total Responses = 35) of PDs at the University of Ottawa agreeing that Leadership Training for their trainees is important. However, there is a gap between buy-in and the delivery of curricula or opportunities for the PGME trainees to develop and apply leadership knowledge and skills. Unfortunately, PDs do not feel prepared to address the majority of CanMEDS leadership key competencies and similarly they report a lack of knowledge of how to develop a leadership curriculum. Currently there is a tremendous variability in the amount of time, structure and focus of leadership training for residents. Similarly, given the vast amount of educational needs and responsibilities for PGME trainees and PDs, it is possible that although leadership training is considered important, its relative importance to other learning needs is not high and thus time may not be adequately dedicated to its development.

In the Approach to Curriculum Development in Medical Education, Kern outlines six-steps that can be utilized to design, implement and evaluate medical education programs. These include:

1. Problem Identification and General Needs Assessment
2. Targeted Needs Assessment
3. Goals and Objectives
4. Educational Strategies
5. Implementation
6. Evaluation and Feedback

Step 1 has been fulfilled by the AFMC, RCPSC and others in that they have identified physician leadership development as a need in Canadian medical training and fortunately, PDs have bought into this concept. At the same time, it is difficult to know if PGME trainees exhibit the same sense of buy-in. Although programs, such as the Residents as Leaders course (RALS), are highly sought after and regarded at the University of Ottawa, only a small portion of residents participate.^8^

The second step in Kern’s process is a targeted needs assessment.^7^ It is suggested that leadership training will require some variability across programs to best address their trainees’ needs, which is congruent with our findings that highlight the variability of opportunities across programs. However, it is uncertain from this study whether PGME trainees have played an active role in helping to design their leadership training. Including PGME trainees in curricula development may be paramount to ensure applicability and buy-in from PGME trainees.

Creating tangible and clear goals and objectives is the third step in Kern’s process.^7^ Although the overarching goal of any leadership training is to develop an effective leader, the concrete description of what makes an effective leader is more elusive. In our study, PDs reported unclear curricular guidelines for leadership training as being significant barriers to leadership training. Currently, the Canadian Medical Association and other societies are utilizing the “LEADS in a Caring Environment”© framework. This framework “defines the knowledge, skills and attitudes a leaders needs to have successfully contribute to an effective, efficient Canadian healthcare system”^9^ and includes five pillars: leading self, engaging others, achieving results, developing coalitions, and transforming systems. Perhaps encouraging programs to utilize this LEADS framework would provide consistency for the trainee throughout his/her career and could encourage him/her to consistently build their skills into an established framework and underscore the need for continued professional development. Similarly, using an established framework will ideally prevent multiple programs and institutions from reinventing goals and objectives, but rather allow them to devote time to creation of resources to develop knowledge and skills in a concerted way.

The development of educational strategies is necessary in curriculum development in medical education and constitutes the fourth steps of Kern’s cycle.^7^ In our study, PDs identified several challenges in this regard, importantly a lack of content expertise and lack of knowledge of other successful PGME leadership programs. Suggestions to overcome these barriers included: sharing and dissemination of educational strategies, support from specialty societies, and a centralization of expertise (and potentially delivery) at the faculty, provincial and/or national levels.

The fifth step of Kern’s six-step approach to curriculum development for medical education is implementation. Implementation of leadership training has been reported as difficult for many PDs.^7^ Scarcity of time and lack of infrastructure have been reported as significant challenges. One PD suggests that perhaps a solution to this barrier is to redefine traditional “service” work as an “opportunity for leadership development”. By providing the PGME trainee with the necessary support, mentorship and resources and investing in residents as leaders during their service roles, the PGME program has the two-fold potential of addressing hospital and patient needs, while at the same time meaningfully equipping residents with the opportunity to apply their skills and knowledge, which will be essential to them as independent practitioners.

Kern’s sixth step in medical education curriculum development focuses on evaluation and feedback, not solely of the trainee but more importantly of the program.^8^ Although the RALS course at the University of Ottawa has been well evaluated and may serve as an example for other programs, there was little to suggest that individual programs’ leadership training elements were evaluated. Similarly, PDs report difficulty in adequately evaluating the PGME trainee in terms of her/his leadership effectiveness.^8^ As further evaluation tools are created, it is paramount that these be disseminated.

### Study limitations

The limited response rate of 55.7% may allow for the results of the study to have been shaped by a non-response bias, thereby undermining the reliability and validity of the survey. Responders were given the option of identifying their program or responding anonymously. As a result, it is impossible to identify any specific trends with respect to the programs that did not respond. Included in the combined group of “non-responders” and “anonymous responders” were the programs of Anesthesia, General Surgery, Obstetrics and Gynecology, which all of have large numbers of trainees. The survey also focuses on PGME leadership training at a single institution and thus does not completely represent the depth and breadth of PGME leadership training in Canada. However, it is likely that most PDs may have knowledge and/or access to PGME leadership strategies through their individual specialty societies. Furthermore, only two semi-structured interviews were conducted. Additionally, qualitative analysis of the interviews was limited to informal assessment for innovative ideas and themes. Notwithstanding these limitations, the findings of this study reflect a gap between the established benchmark for resident leadership and existing training opportunities.

### Conclusions

While there is widespread recognition of the importance of training resident leaders, the nature and design of residency leadership training is highly variable. Our data suggest that stakeholders consider leadership training in PGME valuable; however, there is a scarcity of time and a lack of expertise, resources, and infrastructure to meet the training needs. Similarly, they often do not know how to assess leadership capacity in their trainees. Description of successful leadership training models is a step in addressing this gap and providing PDs with the knowledge they clearly need.

This study presses the need for further research into leadership training for PGME trainees, assessment of the trainees, and also evaluation of leadership curricula. Future strategies may include greater centralization, PGME leadership networks, and clinically oriented leadership curricula.

## Figures and Tables

**Table 1:** Royal College of Physicians and Surgeons of Canada leadership role competencies

Key competencies:	Enabling competencies:
Physicians are able to:	Physicians are able to:
1. Contribute to the improvement of healthcare delivery in teams, organizations and systems.	1.1 Apply the science of quality improvement to contribute to improving systems of patient care 1.2 Analyze adverse events and near misses to enhance systems of care 1.3 Use health informatics to improve the quality of patient care and optimize patient safety
2. Engage in the stewardship of healthcare resources	2.1 Allocate healthcare resources for optimal patient care 2.2 Apply evidence and management processes to achieve cost-appropriate care
3. Demonstrate leadership in professional practice	3.1 Develop their leadership skills 3.2 Design and organize elements of healthcare delivery 3.3 Facilitate change in healthcare to enhance services and outcomes
4. Manage their practice and career	4.1 Set priorities and manage time to balance practice and personal life 4.2 Manage career planning, finances, and health human resources in a practice 4.3 Implement processes to ensure personal practice improvement

**Table 2:** Responding programs and number of trainees

Program	Residents and fellows (*n*)
Anatomical Pathology	15
Cardiac Surgery R/F	12
Child and Adolescent Psychiatry	3
Critical Care R/F	10
Dermatology	18
Emergency Medicine (RCPSC)	60
Endocrinology and Metabolism (S)	10
Family Medicine (CFPC) (P)	168
FM Enhanced Skills (FM) – Palliative Medicine (RCPSC/CFPC) (AWC) (Cat.1)	7
Gastroenterology	10
Geriatric Psychiatry	1
Hematological Pathology R/F	5
Infectious Diseases	4
Internal Medicine	84
Medical Genetics	6
Neonatology (Neonatal-Perinatal) (S) R/F	11
Neurology	31
Neuroradiology	3
Ophthalmology	21
Orthopedic Surgery	42
Otolaryngology - Head and Neck Surgery	13
Pediatric Emergency Medicine	6
Pediatric Neurology	5
Pediatrics	42
Physical Medicine and Rehabilitation	13
Public Health and Prevention Medicine	13
Radiation Oncology	19
Rheumatology	3
Thoracic Surgery R/F	3
Transfusion Medicine (AFC) R/F	0
Urology R/F	22
Psychiatry	62

**Table 3:** Preparedness for CanMEDS 2015 Leadership Role Key Competencies

	Not Prepared	Somewhat Prepared	Prepared	Total Responses
i. Apply the science of quality improvement to contribute to improving systems of patient care	5 (12.8%)	22 (56.4%)	12 (30.8%)	39
ii. Analyze adverse events and near misses to enhance systems of care	5 (12.8%)	21 (53.8%)	13 (33.3%)	39
iii. Use health informatics to improve the quality of patient care and optimize patient safety	11 (28.9%)	17 (44.7%)	10 (26.3%)	38
iv. Allocate healthcare resources for optimal patient care	10 (25.6%)	15 (38.5%)	14 (35.9%)	39
v. Apply evidence and management processes to achieve cost-appropriate care	8 (20.5%)	21 (53.8%)	10 (25.6%)	39
vi. Develop their leadership skills	4 (10.3%)	24 (61.5%)	11 (28.2%)	39
vii. Design and organize elements of healthcare delivery	12 (30.8%)	18 (46.2%)	9 (23.1%)	39
viii. Facilitate change in healthcare to enhance services and outcomes	16 (41.0%)	17 (43.6%)	6 (15.4%)	39
ix. Set priorities and manage time to balance practice and personal life	4 (10.3%)	22 (56.4%)	13 (33.3%)	39
x. Manage career planning	1 (2.6%)	14 (35.9%)	24 (61.5%)	39
xi. Manage personal finances	11 (28.2%)	19 (48.7%)	9 (23.1%)	39
xii. Manage health human resources in a practice	12 (30.8%)	21 (53.8%)	6 (15.4%)	39
xiii. Implement processes to ensure personal practice improvement	5 (12.8%)	21 (53.8%)	13 (33.3%)	39

**Table 4:** Availability of leadership training opportunities: programs

	Mandatory	Optional	Not Presently Offered	Total Responses
CMA/PMI facilitated leadership course	0 (0.0%)	19 (50.0%)	19 (50.0%)	38
CMA/PMI online leadership course	0 (0.0%)	14 (36.8%)	24 (63.2%)	38
University facilitated leadership course	4 (10.8%)	31 (83.8%)	2 (5.4%)	37
Departmental leadership course	1 (2.7%)	16 (43.2%)	20 (54.1%)	37
National society leadership course	1 (2.7%)	18 (48.6%)	18 (48.6%)	37
Leadership focused academic days	13 (34.2%)	9 (23.7%)	16 (42.1%)	38
Simulation course on leadership	2 (5.4%)	6 (16.2%)	29 (78.4%)	37
Multi source feedback programs, i.e., Pulse 360	33 (86.8%)	2 (5.3%)	3 (7.9%)	38
Small group seminars on leadership	6 (15.8%)	11 (28.9%)	21 (55.3%)	38
Resident participation in leadership roles i.e., hospital, departmental, or resident committees	17 (44.7%)	19 (50.0%)	2 (5.3%)	38
Resident leadership initiatives	9 (23.7%)	24 (63.2%)	5 (13.2%)	38
Chief resident course	6 (16.2%)	11 (29.7%)	20 (54.1%)	37

**Table 5:** Availability of leadership opportunities: core skills

	Mandatory	Optional	Not Presently Offered	Total Responses
Self awareness: conscious knowledge of one’s own character, feelings, motives, and desires.	8 (21.1%)	8 (21.1%)	22 (57.9%)	38
Self Reflection: meditation or serious thought about one’s character, actions, and motives.	6 (15.8%)	11 (28.9%)	21 (55.3%)	38
Self Management: management of or by oneself; the taking of responsibility for one’s own behaviour and well-being.	10 (26.3%)	11 (28.9%)	17 (44.7%)	38

**Table 6:** Barriers to implementing leadership training

	Not a Barrier	Moderate Barrier	Significant Barrier	Total Responses
i. Lack of facilitators	6 (16.2%)	23 (62.2%)	8 (21.6%)	37
ii. Lack of buy-in by residents	21 (56.8%)	14 (37.8%)	2 (5.4%)	37
iii. Lack of buy-in by faculty	15 (40.5%)	20 (54.1%)	2 (5.4%)	37
iv. Scarcity of time	3 (8.1%)	11 (29.7%)	23 (62.2%)	37
v. Lack of or limited financial resources	8 (21.1%)	17 (44.7%)	13 (34.2%)	38
vi. Lack of or limited human resources	5 (13.2%)	16 (42.1%)	17 (44.7%)	38
vii. Lack of knowledge of objectives (knowledge/skills) involved in a leadership training curricula	6 (16.2%)	16 (43.2%)	15 (40.5%)	37
viii. Lack of knowledge of how to develop a leadership training curriculum	3 (7.9%)	17 (44.7%)	18 (47.4%)	38
ix. Lack of knowledge of other successful PGME leadership programs	5 (13.5%)	17 (45.9%)	15 (40.5%)	37
x. Lack of evidence to support leadership training	20 (57.1%)	11 (31.4%)	4 (11.4%)	35
xi. Lack of tools to evaluate leadership skills	5 (13.2%)	23 (60.5%)	10 (26.3%)	38
xii. Unclear curricular guidelines for leadership training	3 (7.9%)	20 (52.6%)	15 (39.5%)	38

**Table 7:** Supports for development of leadership training programs

	Not helpful	Somewhat helpful	Very helpful	Total responses
Facilitator training	2 (5.4%)	15 (40.5%)	20 (54.1%)	37
Information about other implemented leadership training curricula	0 (0.0%)	11 (29.7%)	26 (70.3%)	37
Information about the educational/clinical relevance of leadership training	4 (10.8%)	21 (56.8%)	12 (32.4%)	37
Access to educational resources that assist in leadership training	0 (0.0%)	15 (40.5%)	22 (59.5%)	37
Infrastructure to support leadership training	0 (0.0%)	9 (25.7%)	26 (74.3%)	35
Funding to support leadership training	1 (2.7%)	16 (43.2%)	20 (54.1%)	37
Further research to support efficacy of leadership training	12 (32.4%)	20 (54.1%)	5 (13.5%)	37
Protected time for staff for leadership education	3 (8.1%)	9 (24.3%)	25 (67.6%)	37
Tools to evaluate leadership knowledge/skills	1 (2.7%)	12 (32.4%)	24 (64.9%)	37
Access to experts in leadership training	3 (8.3%)	10 (27.8%)	23 (63.9%)	36

## Appendix 1 A Landscape Analysis of Leadership training in Postgraduate Medical Education Training Programs at the University of Ottawa

### Page 1

#### Background

The importance of strong leadership is underscored by the Royal College’s decision to include the “leader” role in the newest iteration of CanMEDs 2015, replacing the former role of “manager.” The Royal College of Physicians and Surgeons of Canada (RCPSC) provides the following definition on leadership,

**“As Leaders, physicians develop a vision of a high-quality health care system and, in collaboration with other health care leaders, take responsibility for effecting change to move the system toward the achievement of that vision.”**

The RCPSC further describes leadership stating:

“Society has explicitly identified leadership and management abilities as core requirements for the practice of medicine. Physicians and others exercise collaborative leadership within the complex health care systems that form their specific work environment s. At a system level, physicians contribute to the development and delivery of continuously improving health care and engage others to work with them toward this vision. Physicians must balance their personal lives with their responsibilities as leaders and managers in their everyday clinical, administrative, research, and teaching activities. They function as individual care providers, as members of teams or groups, and as participants and leaders in the health care system locally, regionally, nationally, and globally. The CanMEDS Leader Role describes the active engagement of all physicians as leaders and managers in decision making in the operation and ongoing evolution of the health care system.”

While, the mandate to train physician leaders is clear, the concrete description of post-graduate leadership curricula and opportunities in postgraduate medical education (PGME) training programs in Canada is not well described and may be challenging for PGME to satisfy. In this survey, which has been distributed to all Program Directors at the University of Ottawa, we are trying to elucidate the current breadth of leadership training/curricula available and the barriers to implementation. The ultimate goal of this project is to provide program directors with resources, curriculum elements and concrete examples of leadership training opportunities so that they may implement them as they see necessary in their own program.

### Page 2

#### CanMEDS 2015

The latest draft of CanMEDS 2015, released in Sept ember 2014, outlines several milestones for the development of the CanMEDS leader role.

**The milestones are listed below. CURRENTLY, how ready is your program to train residents in the following milestones?**

**Table:**

Topic	Not Prepared	Somewhat Prepared	Prepared
i. Apply the science of quality improvement to contribute to improving systems of patient care	○	○	○
ii. Analyze adverse events and near misses to enhance systems of care	○	○	○
iii. Use health informatics to improve the quality of patient care and optimize patient safety	○	○	○
iv. Allocate health care resources for optimal patient care	○	○	○
v. Apply evidence and management processes to achieve cost-appropriate care	○	○	○
vi. Develop their leadership skills	○	○	○
vii. Design and organize elements of health care delivery	○	○	○
viii. Facilitate change in health care to enhance services and out comes	○	○	○
ix. Set priorities and manage time to balance practice and personal life	○	○	○
x. Manage career planning	○	○	○
xi. Manage personal finances	○	○	○
xii. Manage health human resources in a practice	○	○	○
xiii. Implement processes to ensure personal practice improvement	○	○	○

### Page 3

#### Structure and Design of Leadership Training

**From the list below, please indicate what type of leadership opportunities are offered as part of your PGME training program**

**Table:**

Topic	Mandatory	Optional	Not Presently Offered
CMA/PMI facilitated leadership course	○	○	○
CMA/PMI online leadership course	○	○	○
University facilitated leadership course	○	○	○
Departmental leadership course	○	○	○
National society leadership course	○	○	○
Leadership focused academic days	○	○	○
Simulation course on leadership	○	○	○
Multisource feedback programs, i.e. Pulse 360	○	○	○
Small group seminars on leadership	○	○	○
Resident participation in leadership roles i.e. hospital, departmental, or resident committees	○	○	○
Resident leadership initiatives	○	○	○
Chief resident course	○	○	○

**Please describe any additional leadership opportunities offered as part of your training program that were not described in the previous questions**

The current draft of CanMeds 2015 suggests that to achieve resident leadership development involves development of “activities and educational programs that develop self-awareness, self-reflection, and self-management as a leader and a follower in health care organizations.” Please indicate what type of leadership training opportunities are offered as part of your program according to aforementioned competencies.

**Table:**

Topic	Mandatory	Optional	Not Presently Offered
Self awareness: conscious knowledge of one’s own character, feelings, motives, and desires.	○	○	○
Self Reflection: meditation or serious thought about one’s character, actions, and motives.	○	○	○
Self Management: management of or by oneself; the taking of responsibility for one’s own behaviour and well-being.	○	○	○

**How much time per year of residency training is dedicated to leadership training on average?**

Please indicate Not Applicable (N/A) if your residency training program does not include that particular Post Graduate Year (PGY) of training.

**Table:**

Topic	N/A	0	1–5 hours	6–10 hours	11–15 hours	20+ hours
PGY1	○	○	○	○	○	○
PGY2	○	○	○	○	○	○
PGY3	○	○	○	○	○	○
PGY4	○	○	○	○	○	○
PGY5	○	○	○	○	○	○
PGY6	○	○	○	○	○	○

**How is resident leadership training funded within your department?**

Please select all that apply:

□ No funds currently allocated
□ University funding
□ Departmental funding
□ Resident funding
□ External funding
□ Other, please specify

**Who leads resident leadership training?**

Please select all that apply:

□ Departmental faculty
□ Departmental faculty who have undergone leadership training
□ Senior resident s
□ Senior resident who have undergone leadership training
□ External facilitators with specialized training
□ Other, please specify

### Page 4

#### Leadership Training Evaluation

**Is any type of evaluation used to specifically evaluate resident leadership knowledge and/or skills?**

□ Yes □ No

**What form of evaluation does your program use to evaluate resident leadership knowledge/skills?**

Please select all that apply:

□ Direct observation
□ Knowledge test
□ Mini clinical evaluation exercises
□ Multi source feedback exercises
□ Reflective essay
□ OSCEs
□ Simulation
□ Other, please specify

### Page 5

#### Chief Resident s

**Does/do your chief resident (s) receive any formal leadership training?**

□ Yes, please specify what kind of training is provided
□ No
□ Unsure

**Is/are your chief resident (s) formally evaluated on their role as a chief resident?**

□ Yes, please specify what kind of evaluation is provided
□ No
□ Unsure

### Page 6

#### Barriers to Leadership Training

**What are the barriers to implementing leadership training in your program?**

**Table:**

	Not a Barrier	Moderate Barrier	Significant Barrier
i. Lack of facilitators	○	○	○
ii. Lack of buy-in by residents	○	○	○
iii. Lack of buy-in by faculty	○	○	○
iv. Scarcity of time	○	○	○
v. Lack of or limited financial resources	○	○	○
vi. Lack of or limited human resources	○	○	○
vii. Lack of knowledge of objectives (knowledge/skills) involved in a leadership training curricula	○	○	○
viii. Lack of knowledge of how to develop a leadership training curriculum	○	○	○
ix. Lack of knowledge of other successful PGME leadership programs	○	○	○
x. Lack of evidence to support leadership training	○	○	○
xi. Lack of tools to evaluate leadership skills	○	○	○
xii. Unclear curricular guidelines for leadership training	○	○	○

**Please describe any additional barriers to implementing leadership training that were not described in the previous questions**

### Page 7

#### Strategies to Improve Leadership Training

**What supports would be helpful to further develop leadership training/curricula in your PGME program?**

**Table:**

	Not helpful	Somewhat helpful	Very helpful
Facilitator training	○	○	○
Information about other implemented leadership training curricula	○	○	○
Information about the educational/clinical relevance of leadership training	○	○	○
Access to educational resources that assist in leadership training	○	○	○
Infrastructure to support leadership training	○	○	○
Funding to support leadership training	○	○	○
Further research to support efficacy of leadership training	○	○	○
Protected time for staff for leadership education	○	○	○
Tools to evaluate leadership knowledge/skills	○	○	○
Access to experts in leadership training	○	○	○

**Please describe any additional supports that you would find helpful in developing existing leadership training that were not described in the previous questions Please describe any other additional supports (not described in the previous question) that you would find helpful to further develop leadership training/curricula in your PGME program.**

### Page 8

#### The Importance of Leadership Training

**Please indicate what you consider to be the level of importance of resident participation in leadership training.**

□ Unimportant
□ Neither Unimportant or Important
□ Important

**What do you see as the role of resident leadership in your training program?**

**If possible, please describe a success story involving resident participation in leadership training in your program?**

**In your program, has there been any impact resulting from resident participation in leadership training? If so, please describe.**

**Your PGME trainees will likely be the healthcare leaders of tomorrow. What would you like to see implementd to ensure that they are better prepared to engage in healthcare transformation?**

### Page 9

#### Thank You For Participating In This Survey

**Please indicate your PGME training program. This information is needed to prevent you from receiving reminders.**

Adult Cardiac Electrophysiology
Adult Echocardiography
Adult Interventional Cardiology
Anatomical Pathology
Anesthesiology
Cardiac Surgery
Cardiology
Child and Adolescent Psychiatry
Clinician Investigator Program
Colorectal Surgery
Critical Care Medicine
Dermatology
Diagnostic Radiology
Emergency Medicine
Endocrinology and Metabolism
Family Medicine
FM Enhanced Skills – Care of the Elderly
FM Enhanced Skills – Clinician Scholar
FM Enhanced Skills – Emergency Medicine
FM Enhanced Skills – Family Practice Anesthesia
FM Enhanced Skills – Palliative Medicine
Forensic Psychiatry
Gastroenterology
General Internal Medicine
General Pathology
General Surgery
Geriatric Medicine
Geriatric Psychiatry
Gynecologic Oncology
Gynecologic Reproductive Endocrinology and Infertility
Hematological Pathology
Hematology
Infectious Diseases
Internal Medicine
Maternal / Fetal Medicine
Medical Genetics
Medical Microbiology
Medical Oncology
Neonatology (Neonatal-Perinatal)
Nephrology
Neurology
Neuroradiology
Neurosurgery
Nuclear Medicine
Obstetrics & Gynecology
Ophthalmology
Orthopedic Surgery
Otolaryngology - Head & Neck Surgery
Pediatric Cardiology
Pediatric Critical Care Medicine
Pediatric Emergency Medicine
Pediatric Endocrinology and Metabolism
Pediatric Hematology / Oncology
Pediatric Infectious Diseases
Pediatric Nephrology
Pediatric Neurology
Pediatric Radiology
Pediatric Surgery
Pediatrics
Physical Medicine and Rehabilitation
Plastic Surgery
Psychiatry
Public Health and Preventive
Medicine
Radiation Oncology
Respirology
Rheumatology
Thoracic Surgery
Transfusion Medicine
Urology
Vascular Surgery

**If we identify your program as having an excellent element of leadership training/curriculum, would you be willing to be contacted for further discussion?**

□ Yes, please indicate the preferred email address for correspondence
□ No

Comments and suggestions:

## Appendix 2

**Table 8:** Summary of qualitative findings and recommendations

1. Leadership training is important	PDs overwhelmingly agree that leadership training is important to resident training (Figure 1).
2. PDs would like PGME leadership training support	PDs want to learn more about what other programs are doing to gain assistance in developing their current programs. PDs are seeking assistance with developing, implementing and evaluating leadership training programs (Table 6).
3. PDs would like to be aware of and have access to national and international Leadership Training opportunities for their Trainees	Some PDs take advantage of leadership opportunities offered nationally and internationally. Some PDs encourage chief residents and other appointed/elected leaders in their program to attend external leadership training. Attendance of resident leaders to external symposia was helpful in allowing them to gain training from outside experts and in turn share this training upon returning, and simultaneously reduce the curricular burden on home programs.
4. PDs would like REAL Leadership opportunities to be made available to their Trainees	PDs are in favour of incorporating resident leadership as a part of their program and hospital culture. One PD described how, in their movement to further empower residents, residents were made site chiefs at the various hospitals. Another program outlined how residents are incorporated into academic and hospital committees. Making residents a part of the hospital leadership culture, as noted by this PD, has been instrumental in convincing faculty to support resident attendance in formal leadership development activities.
5. PDs would like their University to centralize some PGME Leadership Training for access by all programs.	Some PDs encouraged resident participation in local leadership programs and had history of strong resident attendance in such programs. PDs overwhelmingly support the “Resident as Leaders Program” (RALs) course, a five-day face-to-face course followed by a longitudinal leadership practical experience offered to residents at the University of Ottawa (9). Interested residents must be supported by their residency program and in turn be accepted to the program.
6. PDs would like to redefine SERVICE to LEADERSHIP as a means of highlighting and developing practical application of leadership knowledge and skills and provide the support and mentorship needed to do so	An important theme that emerged in interviews with PDs was the relationship between resident *service* and leadership opportunities. Resident *service*, namely the clinical responsibilities required during residency, can be skilfully integrated with resident leadership opportunities, as described by one PD. When coupled with mentorship and teaching, investing in residents as leaders during their service roles has the two fold potential of addressing hospital and patient needs, while at the same time meaningfully equipping residents with skills and experience which will be essential to them as independent practitioners.
7. Information Sharing	PDs highlighted the need for greater communication between residency programs. PDs are interested in learning what other programs are doing and how they can adopt such opportunities into their program. One prominent success story of information sharing is the RALs leadership program, which is open to all residency programs at the University of Ottawa. PDs voiced a need for further clarity from the Royal College with respect to specific benchmarks for leadership competencies (Table 6).
8. PDs feel there should be Recognition Trainees for Leadership successes	PDs recognize resident involvement in leadership training has been a critical driving force of department and hospital improvements. One PD shared, “Leadership in a safety initiative by a previous resident has tangibly improved patient care and provided a career path for that resident.” Another PD noted, “…residents who took the faculty leadership course have become important drivers of positive change in our department.” In addition to the impact on the department/division and hospital, resident involvement in leadership has also been essential for professional development. One PD noted, “A former resident that underwent training became the Quality and Safety lead in the Division, and shows potential to be [a future] Division chief.”
